# Comparison of models for stroke-free survival prediction in patients with CADASIL

**DOI:** 10.1038/s41598-023-49552-w

**Published:** 2023-12-17

**Authors:** Henri Chhoa, Hugues Chabriat, Sylvie Chevret, Lucie Biard

**Affiliations:** 1 ECSTRRA Team, Université Paris Cité, UMR1153, INSERM, Paris, France; 2 Centre NeuroVasculaire Translationnel - Centre de Référence CERVCO, DMU NeuroSciences, Hôpital Lariboisière, GHU APHP-Nord, Université Paris Cité, Paris, France; 3 INSERM NeuroDiderot UMR 1141, GenMedStroke Team, Paris, France

**Keywords:** Risk factors, Epidemiology, Neurology, Cerebrovascular disorders

## Abstract

Cerebral autosomal dominant arteriopathy with subcortical infarcts and leukoencephalopathy, which is caused by mutations of the NOTCH3 gene, has a large heterogeneous progression, presenting with declines of various clinical scores and occurrences of various clinical event. To help assess disease progression, this work focused on predicting the composite endpoint of stroke-free survival time by comparing the performance of Cox proportional hazards regression to that of machine learning models using one of four feature selection approaches applied to demographic, clinical and magnetic resonance imaging observational data collected from a study cohort of 482 patients. The quality of the modeling process and the predictive performance were evaluated in a nested cross-validation procedure using the time-dependent Brier Score and AUC at 5 years from baseline, the former measuring the overall performance including calibration and the latter highlighting the discrimination ability, with both metrics taking into account the presence of right-censoring. The best model for each metric was the componentwise gradient boosting model with a mean Brier score of 0.165 and the random survival forest model with a mean AUC of 0.773, both combined with the LASSO feature selection method.

## Introduction

In clinical studies, survival models are built to understand and predict event occurrences^1^. This is particularly important for cerebral autosomal dominant arteriopathy with subcortical infarcts and leukoencephalopathy (CADASIL), the most frequent hereditary cerebral small vessel disease^2^. The disease is caused by stereotyped mutations of the NOTCH3 gene, which encodes a transmembrane receptor of vascular smooth muscle cells and pericytes. These mutations lead to an odd number of cysteine residues within the epidermal growth factor repeats of the receptor. They result in a progressive accumulation of NOTCH3 extracellular domains (NOTCH3-ECD) aggregating with multiple matrix proteins in the wall of arterioles and capillaries^3,4^.

However, there is a large variability in clinical manifestations and level of disability among CADASIL patients^5,6^. It is therefore particularly important to develop prediction models to better specify the prognosis of this rare disease and to prepare therapeutic trials in the future. Some studies have suggested that sex, cardiovascular risk factors, such as smoking or hypertension, and other disease characteristics, might influence the clinical expression of the disease^6^. For example, a multivariable model to predict 3-year variation in the Mattis Dementia Rating Scale (MDRS) score has been proposed^7^. This previous model is based on demographic (sex and age), clinical (modified Rankin score^8^, balance problems, and gait disturbances), and imaging variables (white matter hyperintensities (WMHs), number of lacunes^9,10^, microbleeds^11^, and brain parenchymal fraction^12,13^).

Among all possible manifestations of the disease, the occurrence of stroke or death are severe outcomes^5,14^. Hence, it is essential to forecast these events to develop early preventive measures. In the present study, we aimed to build the best predictive model of stroke and death events using linear and nonlinear models and several feature selection approaches.

Different types of models have been proposed that predict the risk of experiencing an event by using multiple predictor variables. The traditional Cox proportional hazards (CPH) regression^15^, which is certainly the most commonly used, has been modified to increase its generalizability by adding a penalization term, leading to several derivatives, namely, the ridge Cox regression, the LASSO Cox regression or the elastic net Cox regression which apply $L2$, $L1$, and both $L2$ and $L1$ penalization terms, respectively^16–18^. Furthermore, machine learning-based algorithms^19^ have been developed that improve performance by accounting for nonlinear relationships, for example, the survival trees algorithm^20–22^, or by exploiting ensemble methods as in the random survival forest or even gradient boosted models^23,24^. These models have been adapted to be used with survival data, as they were known to have excellent performance on prediction tasks^25,26^.

At the same time, feature selection is an integral part of building a well-performing model. Finding the best predictive features and selecting the right number of predictors enhances both the predictive performance of the model and its generalizability^19,27,28^. Indeed, having too many redundant features can easily lead to overfitting, and the resulting model may behave poorly when confronted with new data. On the other hand, discarding too many features can lead to underfitting and poor performance because of the lack of training data^29^. Feature selection methods can be classified into three groups^27^. Filter methods examine the relationships of a feature and the target variable to understand the importance of that feature^30^. Wrapper methods generate many models with different subsets of input features and select the features that result in the best performing model according to a performance metric^31^. Finally, embedded methods refer to algorithms that perform feature selection as a part of the learning process to minimize overfitting, notably in the presence of highly correlated predictors^32^. Examples of these are tree-based models, which select the best feature to split at each node, and LASSO, which sets a forced number of features to zero depending on the regularization intensity^32^.

Finally, the entire modeling process can be evaluated by a nested cross-validation (CV) procedure, where the model predictive performance evaluation on out-of-sample data reflects the quality of the feature selection process, the hyperparameter tuning phase, and the model itself^33^. The performance metric should be appropriately chosen depending on the objective. For discrimination purposes, ranking metrics that allow us to handle censored cases such as Harrell’s C-index (i.e., the fraction of pairs for which the event order matches the order of linear-predictor values) and time-dependent area under the curve (AUC) are commonly used. The time-dependent Brier score is better known to assess the calibration and overall performance of a model^34^.

Such approaches, considering the development of predictive models in a high-dimensional setting, have not yet been applied to the CADASIL population. Hence, in this study, we proposed a comparison of the calibration and discrimination predictive performance of distinct survival models for stroke-free survival prediction in CADASIL patients, using different feature selection approaches with the CPH model and machine learning-based algorithms.

## Results

### Performance of the models and feature selection approaches based on the nested cross-validation

**Figure 1:**
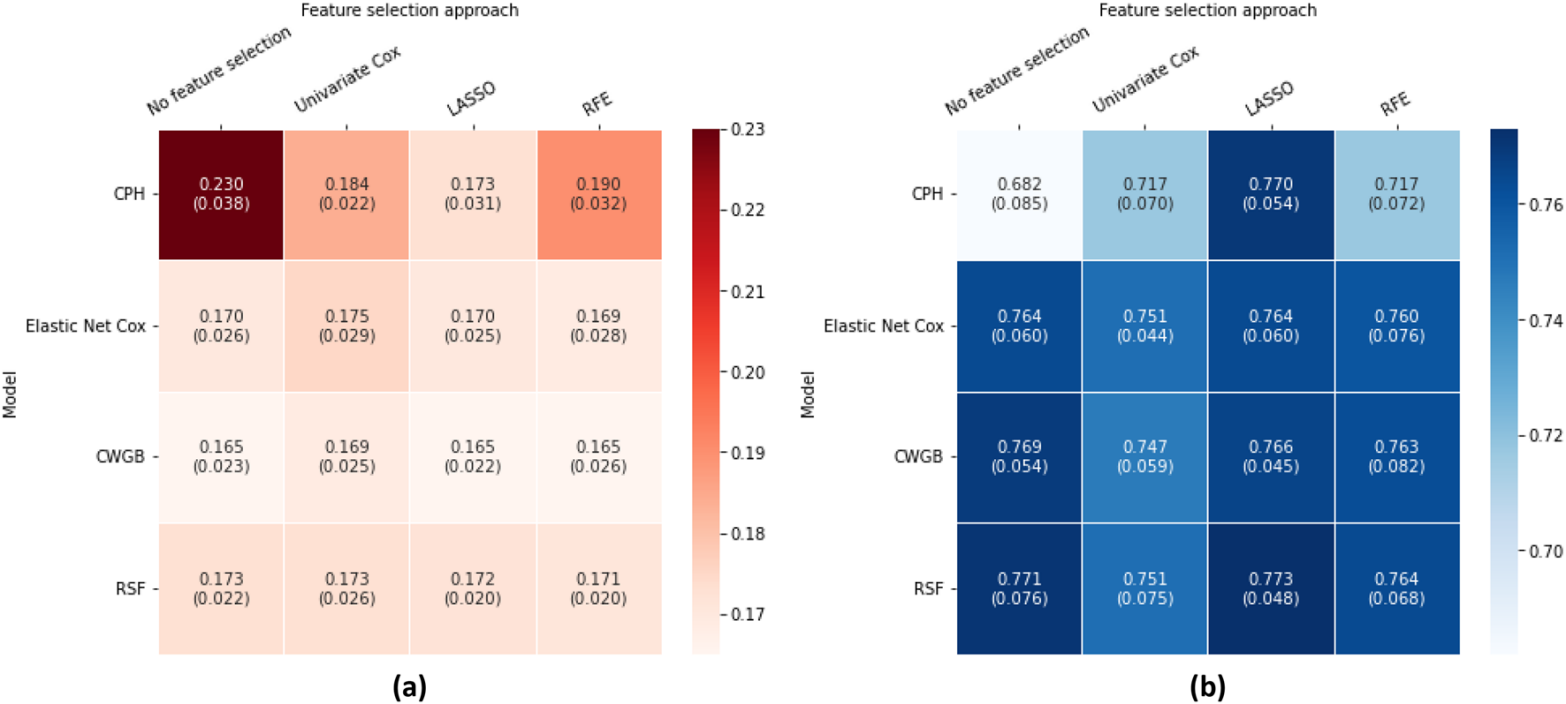
Heatmap illustrating the nested CV results with the mean and standard deviation of the performances for each model and feature selection approach pair, measured by the time-dependent Brier Score in (**a**) and AUC in (**b**) at 5 years from baseline.

As shown in Fig. 1, there was no single model that dramatically outperformed the others in the nested cross-validation (CV), but some noticeable differences were observed between the feature selection approaches. Indeed, the LASSO method was the overall best method, as it produced the best results for almost all of the models, as well as being used in the global best models as defined by each of the two metrics. Specifically, when using LASSO, the componentwise gradient boosting (CWGB) algorithm outperformed the other models when considering the best overall performance, with a time-dependent Brier score at 5 years of 0.165 and a standard deviation of 0.022. Additionally, the random survival forest (RSF) algorithm with LASSO feature selected achieved the best discrimination score with a mean time-dependent AUC at 5 years from baseline of 0.773 and a standard deviation of 0.048.

In contrast, the CPH benchmark model showed worse performance than that of all the other models according to both metrics and with every feature selection approach, except for the LASSO method, which led to comparable performance with the other models. Both metrics also indicated it was the worst overall model by a large margin when no feature selection was performed before model fitting. Globally, the CWGB algorithm performed better than the other models when optimizing the overall performance and calibration with the time-dependent Brier Score metric at 5 years from baseline, whereas the RSF model seems to outperform the other models in discrimination when considering the time-dependent AUC metric.

Apart from the performance of models with feature selection with the LASSO method, not performing feature selection before model fitting led to better results for all machine learning-inspired models. Among the three feature selection techniques, univariable Cox scoring performed the worst every single time. On the other hand, the recursive feature elimination algorithm performed quite similarly to the LASSO method on average but exhibited less stability, as shown by the higher standard deviations in each case.

### Final model

As we determined the best models according to each metric, namely the CWGB for the time-dependent Brier Score and the RSF for the time-dependent AUC, we fit the final models with all 99 data features available (list available in Supplementary Table S1) and used the set of hyperparameters that optimized a tenfold CV run (see Supplementary Table S2) with the LASSO feature selection method. The best hyperparameters resulting from the tenfold CV for the CWGB reached a mean Brier score of 0.165 and a standard deviation of 0.023, and the RSF reached a mean AUC of 0.788 and a standard deviation of 0.058.

**Figure 2:**
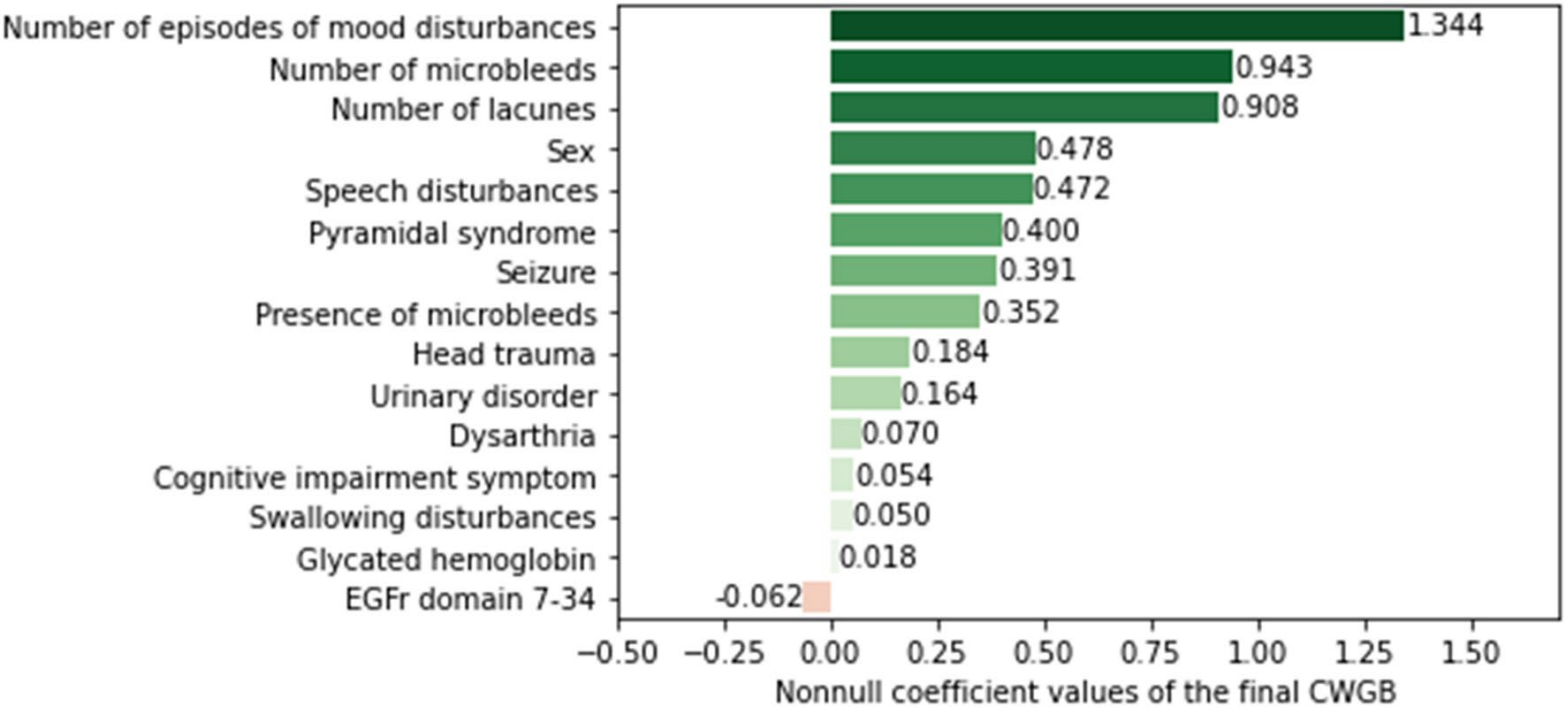
Componentwise gradient boosting nonnull coefficients in decreasing order.

**Figure 3:**
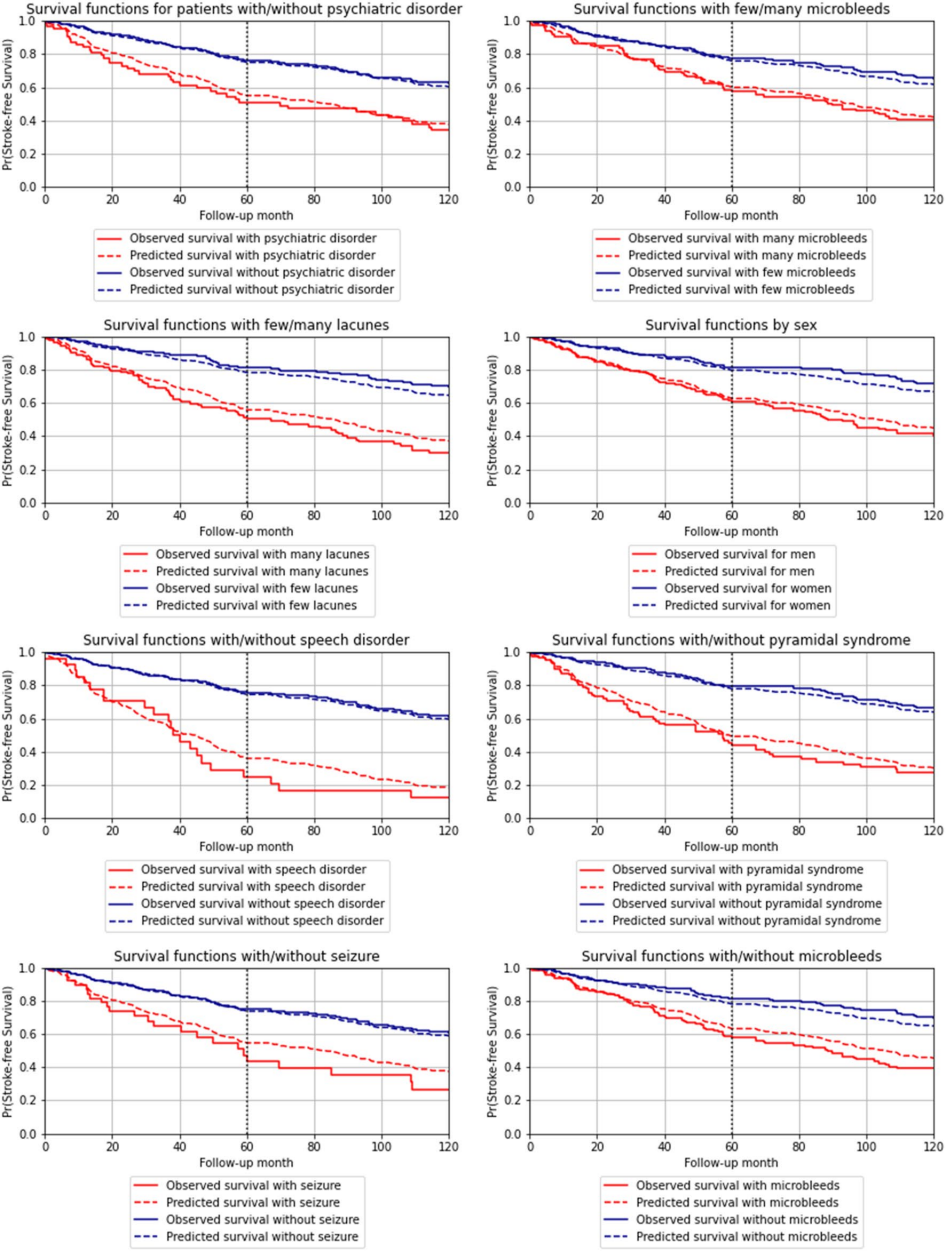
Comparison of the observed and predicted survival functions for the eight strongest features of the componentwise gradient boosting (CWGB) model.

The 15 nonzero coefficients of the CWGB model are represented in Fig. 2. These 15 features were selected by the CWGB algorithm, with the number of episodes of mood disturbances, microbleeds and lacunes having the larger positive effects on the risk prediction and an EGFr mutation location in domains 7 to 34 being the only feature with a negative effect on the risk. The predicted and observed survival functions obtained with the CWGB algorithm and the Kaplan‒Meier estimator are shown in Fig. 3 for the eight strongest features identified by the CWGB algorithm. This visualization of the survival curves exhibits their closeness when stratifying by the features and was expected based on the good calibration performance of the model. For instance, the 5-year predicted stroke-free survival probability was 55.1% (observed 50.8%) in CADASIL patients with episodes of mood disturbances versus 75.1% (observed 76.0%) in those without, and from 74.1% (observed 78.5%) in patients with an EGFr mutation location in domains 7 to 34 versus 71.0% (observed 68.9%) in those with an EGFr mutation location in domains 1 to 6.

Lastly, we did not provide the coefficients for the Random Survival Forest (RSF) because of its non-parametric nature, as it does not assume a specific functional form of the relationship between predictors and survival time. Hence, it would not be appropriate to estimate the marginal effect of individual variables across all trees on the survival predictions.

## Discussion

Using the many features observed in this large CADASIL study cohort, we developed two survival models depending on the desired objective, to develop both an overall well-performing model in terms of calibration and discrimination and a more discrimination-specific model, namely, based on componentwise gradient boosting and random survival forest models. As not all observed patient characteristics are useful for predicting survival, we compared several feature selection approaches to increase performance and generalizability, with LASSO being the best method overall. The quality of the modeling process was assessed using a nested cross-validation (CV) procedure to effectively tune the hyperparameters and produce an unbiased estimation of the model performance on unseen data at the same time.

As shown by the nested CV results, the machine learning algorithms outperformed the Cox proportional hazards (CPH) model for nearly all features selection methods, except the LASSO method. Intuitively, the RSF performs better in discrimination because its model fitting process involves successive splitting rules that optimize the discrimination of the individuals based on their survival. On the other hand, the CWGB excels in calibration since the fitting process consists in minimizing the likelihood loss function, leading to more precise survival predictions at each weak learner addition. Overall, both RSF and CWGB models perform well because they are ensemble models with many hyperparameters to prevent overfitting, which makes them more robust and powerful than individual-based models. When no feature selection was applied beforehand, the CPH had considerable worse performance than the other models, as it was not designed to handle high-dimensional data. In addition, this is likely explained by the fact that machine learning models inherently use embedded feature selection by construction, so having a larger set of features when building the model might result in better fitting and prediction than performing the feature selection separately. Even the LASSO feature selection yields only a small improvement in the performance compared to this approach.

For machine learning models, the univariable Cox scoring feature selection method led to the worst results, which might be explained by the fact that this method is univariate and thus does not take into account any potential interactions between the features, only considering the individual predictive power of each feature. Furthermore, even though the recursive feature elimination (RFE) algorithm led to quite good model performance, this method was still outperformed by LASSO, and its greater computation time along with its greater performance instability makes it less desirable.

Based on these models, predictors of an increased risk of shortened stroke-free survival were the number of episodes of mood disturbances, microbleeds and lacunes, while an EGFr mutation location in domains 7 to 34 appeared to delay the occurrence of those events. The high predictive value of lacunes and microbleeds might reflect that these lesions, conversely to white-matter hyperintensities or enlarged perivascular spaces, only occur in presence of advanced structural alterations in the microvessel wall. Particularly, lacunes may occur in CADASIL when damage or loss of smooth muscle cells is such that the vessel can no longer ensure minimal irrigation for cell survival in focal deep cerebral areas^35^. Microbleeds may also result from severe focal parietal modifications with loss of vascular wall integrity allowing the passage of red blood cells in the cerebral tissue^36^. Conversely, the protective effect of EGFR mutations in domains 7 to 34 could be related to a lower accumulation of NOTCH3-ECD in the vascular wall associated with these variants^14^. Whether this lower accumulation, compared to that observed with mutations in EGFR domains 1 to 6, result from a lower exposition to extracellular matrix proteins that co-aggregate^37–39^ with NOTCH3-ECD or from the larger distance of the variant from the cleavage site of NOTCH3 between EGFr 1 and 2, remain undetermined^7,37^. Overall, our findings are consistent with current knowledge on CADASIL disease and factors related to patients’ phenotype and outcomes^38–41^. Our contribution relies on the use of innovating analysis methods allowing identifying independent predictors in high-dimensional settings. These statistical methods are especially relevant in the context of rare diseases and could be applied to other diseases than CADASIL.

As the feature selection method played an important role in the model performance, as shown in the results of the nested CV procedure, several other techniques might be interesting to explore, such as the forward/backward selection algorithm or the permutation importance method^42^. The former iteratively builds the best subset of features by adding/removing features that contribute the most/least to the model’s performance. The latter involves training a model with all features and then systematically shuffling the values of each feature one at a time to observe the impact on the model's performance. The greater the drop in performance when a feature’s values are permuted, the more important it is considered to be. Alternatively, it may be relevant to use all these techniques and keep the features selected by most methods^19^.

The nested CV procedure induced a significant amount of computational complexity, so we were limited in the choice of the hyperparameter search grid. More time and computational power would allow for a more detailed search, which is especially useful for implementing the random survival forest model, which contains several hyperparameters. Additionally, the random splitting of the data in the nested CV induced variability in our results. Even though by a repeated nested cross-validation, here again, the increased computational cost prevented us from doing so.

Missing data were handled using simple imputation with the median for numeric features and the mode for categorical features. More elaborate methods could have been used, such as iterative imputation. Indeed, if we assume that data are missing at random (MAR), it is possible to predict a missing value given the other feature values. Thus, in an iterative fashion, the missing values of a feature are updated by fitting a model on the missing features against the others and predicting the missing values until the model converges. When this method is applied several times, leading to multiple imputed datasets, it is known as multiple imputation. However, this method drastically increases the computation time, so we did not implement this idea here, even though it has been proven to be potentially effective in mitigating the impact of missing data.

Another technique that can be explored to optimize model performance is model stacking^43^. The predictions made by different base models are used as the input features of a meta model, generally a simple one, which generates the final prediction. For example, we could build the first level of models with a random survival forest, a survival tree and a ridge Cox regression model and use their predictions as input to a Cox proportional hazards regression to compute the final predictions. Although this technique is known to have good performance in prediction tasks because it uses the quality of each base estimator prediction, it adds a great amount of complexity by combining several different models, which themselves add many hyperparameters to tune for optimal performance.

Finally, our models only used observational data recorded at baseline. However, as the study cohort is followed over time, it might be interesting and useful to incorporate new data to make use of all the available information. For example, creating new features by summarizing the measures over time can add information to the model. Alternatively, joint modeling that combines the time to an event and the time-varying covariates is another possible area of research^44^. Moreover, we considered only the first occurring event after baseline, but it may also be important to consider the occurrence of several strokes or even the distinct occurrence of stroke and death with different types of models more appropriate for such a task^45^. Some models have been proposed to handle longitudinal data with competing risk in deep learning, for instance, but the amount of data to which we had access is not sufficient for this approach despite its promising results in other applications^46^.

Despite several limitations and possible changes to the approaches we used, our results provide relevant information on baseline predictors of outcomes in CADASIL patients, using high-dimensional statistical analyses. Such a prediction approach is most useful to clinicians and clinically relevant to patients, providing information available at time of diagnosis regarding the prediction of their outcomes. Moreover, the proposed analysis method could be fine-tuned but it is unlikely that, based on the same set of candidate baseline features, results would be drastically different.

## Methods

### Characteristics of the study sample

A total of 482 patients aged over 18 years who were diagnosed with CADASIL were enrolled between June 03, 2003 and December 29, 2020 from the French National Referral Center for rare cerebrovascular diseases in France (www.cervco.fr). CADASIL diagnosis was confirmed by genetic testing (blood sample) results showing a typical cysteine mutation of the NOTCH3 gene.

This study was approved by an independent ethics committee (updated agreement CEEI-IRB-17/388) and conducted in accordance with the Declaration of Helsinki and guidelines for Good Clinical Practice and General Data Protection Regulation (GDPR) in Europe. Informed consent was obtained from all individual participants included in the study.

Our event of interest, or target variable, was the right-censored composite endpoint of the time to either stroke or death, whichever occurred first, or in other words, stroke-free survival. More precisely, in our study, we only considered ischemic and hemorrhagic strokes, as opposed to transient ischemic attacks (TIAs). Indeed, these are less severe forms of stroke, so we did not think it was appropriate to consider TIAs on the same level as major strokes and death.

**Figure 4:**
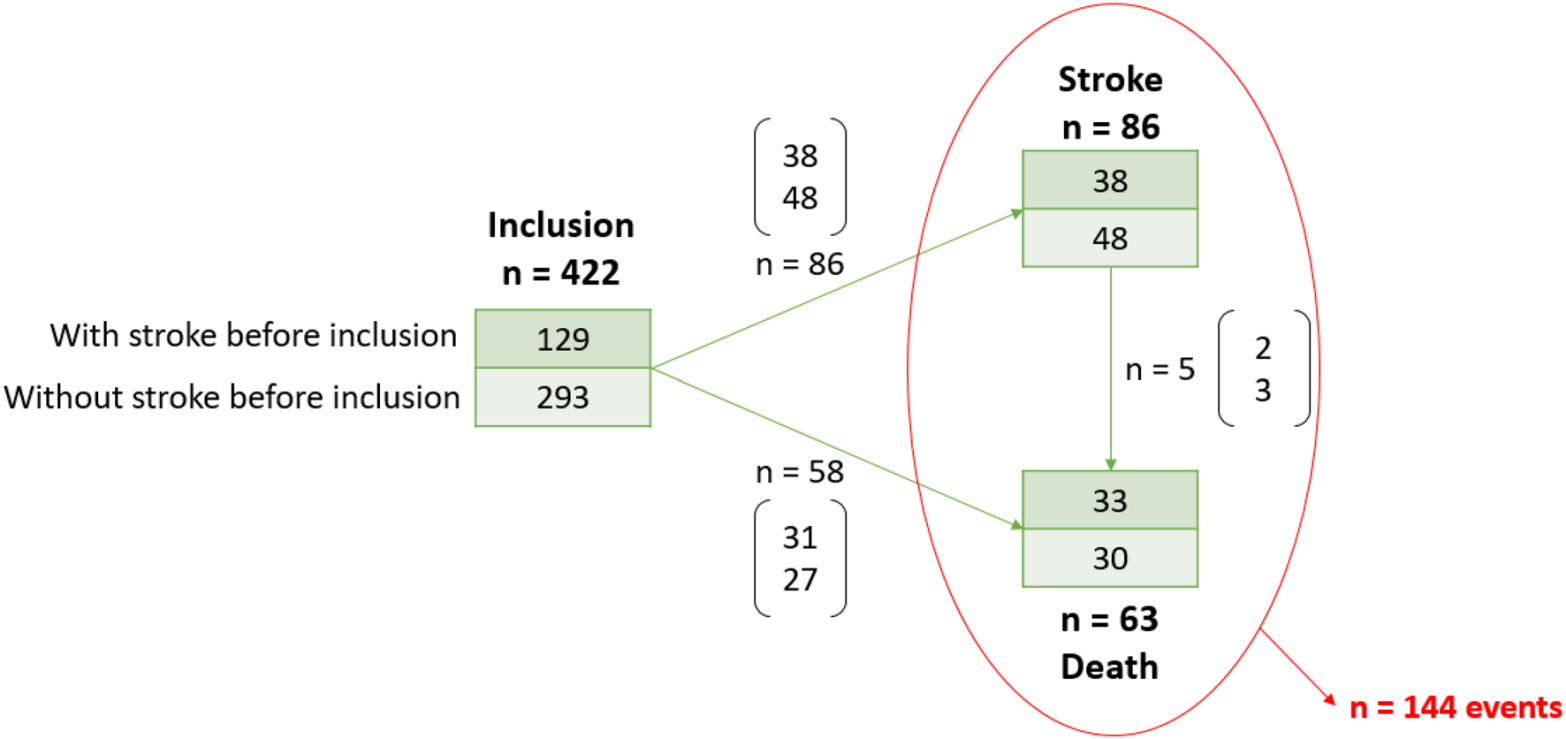
Flowchart of the patients and the events occurrences, with the distinction between the patients having experienced a stroke before inclusion or not.

Of the 482 patients included at baseline, 60 had no follow-up and thus provided had no information about having experienced any event. Of the 422 remaining patients, 129 (31%) developed a stroke before inclusion and 293 (69%) did not. During the follow-up, 86 had a stroke, five of them subsequently died, and 58 patients died without any stroke, thus accounting for a total of 144 events. A detailed summary can be found in the flowchart in Fig. 4.

### Measurements

Clinical data were collected prospectively during individual consultations by board-certified neurologists using a standardized questionnaire. They included several clinical indexes that were systematically recorded in each individual at cohort entry: the Barthel index^47^ ranging from 0 (total patient dependence) to 100 (normal) and the Modified Rankin scale (mRS)^8^ ranging from 0 (normal) to 6 (death), and, to assess cognitive impairment, the index of the Mini-Mental State Examination (MMSE)^48^ ranging from 0 (severe cognitive impairment) to 30 (normal cognition). Several neurological, cognitive and functional test scores were included as well, such as the Mattis Dementia Rating Scale score^49^, the VADAS-COG score^50^, the Trail Making Test (TMT) A and B time^51^, various memory scores derived from the Grober and Buschke test^52^, etc. Furthermore, patients were assessed for the existence of stroke clinical manifestations, for example, dementia or disability related to gait disturbances, urinary disturbances or swallowing difficulties. We also recorded parameters obtained from brain magnetic resonance imaging (MRI), namely, the number of lacunes, defined as small cavities with a rounded pattern and a diameter of less than 15 mm, hyperintensities on T2-weighted and hypointensities on T1-weighted images and distinct from dilated perivascular spaces^9,10^, the number of microbleeds, defined as rounded hypointensities with a diameter less than 10 mm on susceptibility-weighted images^11^, and the brain parenchymal fraction (BPF), for which the brain volume was calculated based on 3D-T1-weighted and normalized with the volume of the intracranial cavity on images using SIENAX methods^12,13^. Finally, some biological data were recorded via biological sampling, such as the hemoglobin, triglycerides, or low-density lipoprotein (LDL) and high-density lipoprotein (HDL) cholesterol. All the features are listed in the Supplementary Table S1.

### Model selection

Four survival models and four feature selection approaches allowing handling high-dimensional right-censored data were considered in our study to model stroke-free survival. For each combination of model and feature selection method, we recorded the prediction quality with respect to two performance metrics, resulting in 16 different performance results for each metric.

The models considered were the Cox Proportional Hazards (CPH) model^15^, which acts as the benchmark model, the Elastic Net Cox Regression^17^, which incorporates regularization techniques to handle more efficiently high-dimensional data, the Component-Wise Gradient Boosting (CWGB) model^53^, and the random survival forest (RSF) model^23^, which are both machine-learning ensemble models with several hyperparameters to prevent overfitting and to effectively capture the data complex relationships. A brief description of these models can be found in the Supplementary Methods.

The feature selection methods consisted of a filter method, the univariate Cox scoring rule^19^, an embedded method, LASSO Cox regression^54^, and a wrapper method, the recursive feature elimination^55^ (RFE) algorithm. Moreover, not performing external feature selection before model building was also considered as the reference approach. A brief description of these methods can be found in the Supplementary Methods.

Finally, to assess the predictive performance of the modeling process, we used the time-dependent Brier Score and the time-dependent AUC, which are two metrics suitable for right-censored data^34^. The former evaluated the overall performance, including calibration, whereas the latter focused on the discrimination ability of the models. A brief description of these metrics can be found in the Supplementary Methods.

### Nested cross-validation

Since a model’s performance is strongly influenced by its hyperparameters, we needed to define a procedure to optimize the hyperparameters with respect to a given metric and at the same time find an unbiased way to evaluate the model performance for feature selection. A common way to handle this problem is to create a nested cross-validation (CV) procedure which involves two nested loops of CV. The outer loop aims to estimate the predictive performance and the inner loop aims to find the best set of hyperparameters that optimizes the performance metric. In our case, we decided to consider two performance metrics, the time-dependent Brier Score and the time-dependent AUC, resulting in two final optimal models, one for each metric. We refer the reader to Fig. 4 which illustrates schematically the nested CV procedure. The entire dataset was split into 10 folds for the outer loop, thus resulting in one fold for model evaluation and nine folds for training. Within a training set, another split in four folds was performed, such that, for each set of hyperparameters, three folds were used to fit the model and the last one for assessing the model performance. As a result, the inner CV produced four values of the performance metric that were averaged to determine the best set of hyperparameters. In turn, these optimal hyperparameters were then used to fit the whole training set of the outer loop. Finally, the fitted model was evaluated on the holdout set of the outer loop. The summary statistics of the model performance were obtained by averaging the ten values of the predictive performance metric resulting from the outer CV and by computing their standard deviations.

After completing the nested CV procedure, for each candidate model, the model with the best performance was selected and we fit this final model to make use of all the available data. To do so, we applied the optimal hyperparameter search in a standard tenfold CV. Then, the set of hyperparameters that led to the best mean performance across the tenfolds is used to fit the model one last time on the whole dataset.

**Figure 5:**
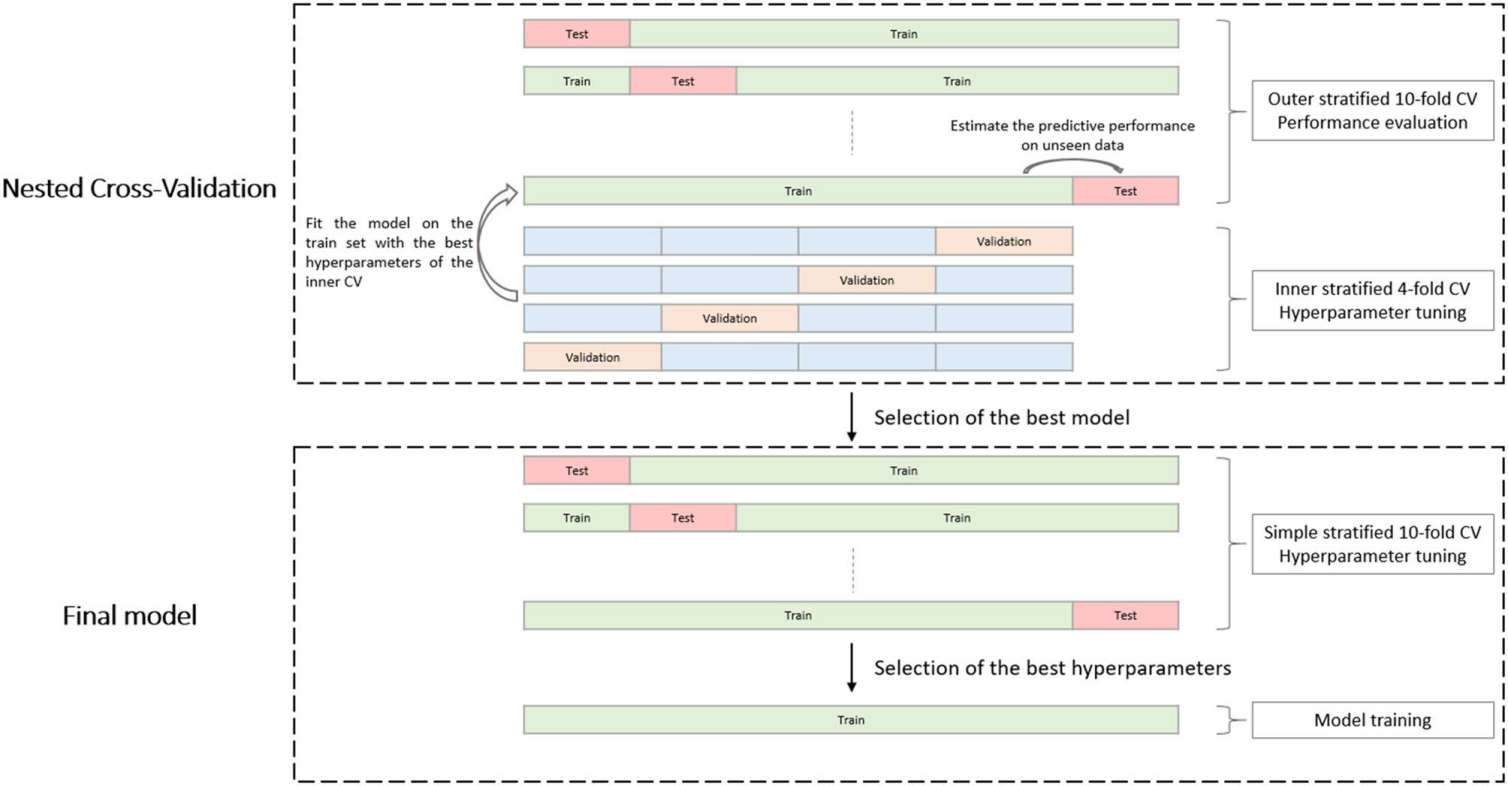
Nested cross-validation for hyperparameter tuning and evaluation of the modeling process followed by final model building.

In order to minimize any potential additional bias from the splitting, the data was randomly shuffled beforehand, and the outer tenfold and inner fourfold CV were stratified with respect to the occurrence of the event of interest. In the end, we obtained two final models after two nested CV runs, one maximizing. The performance in terms of the time-dependent Brier Score metric and the other one optimizing the time-dependent AUC. The detailed procedure is summarized in Fig. 5, and the functions with their hyperparameters used in this method are detailed in Supplementary Table S3.

### Data preprocessing

Data preprocessing was carried out to transform our data into an appropriate and useful form for the models to extract as much information as possible.

Any features with more than 50% their values missing were discarded. The remaining missing data were handled using simple imputation. Categorical variables were imputed with the mode (most frequent category) computed solely from the training set. For numeric variables, missing values were imputed with the median value from the training data. Subsequently, numeric features were scaled to ensure that their values fell within the [0;1] range, using their respective maximum and minimum values computed from the training set. By applying these techniques using only training information, we prevented any data leakage from the test set. Furthermore, constant features across all observations and binary variables with class imbalance greater than 95% were removed from the analysis.

For censored observations, the last observable survival time was computed as the time to the latest event between the last follow-up visit and the last phone call check-up. Finally, the data were truncated at 10 years from baseline, and duplicated observations were omitted.

Following data preprocessing, the final dataset was composed of 99 features, of which 46 were binary and 53 were numeric.

All code for the experiments and the visualization was written in Python.

### Supplementary Information

Supplementary Information.

## Data Availability

The data that support the findings of this study are available on request from the corresponding author.
