# Supplementary material for: Comparison of models for stroke-free survival prediction in patients with CADASIL
Source: Sci Rep. 2023 Dec 17;13:22443. doi: 10.1038/s41598-023-49552-w (PMC10725863; doi:10.1038/s41598-023-49552-w)
Supplement: Supplementary file 1 — Supplementary Information. [file 41598_2023_49552_MOESM1_ESM.pdf]

Comparison of models for stroke-free survival prediction in patients with CADASIL

Supplementary Material

Henri Chhoa[1], Hugues Chabriat[2,3], Sylvie Chevret[1], Lucie Biard[1]

[1]ECSTRRA Team, Université de Paris, UMR1153, INSERM, Paris, France

[2]Translational Neurovascular Centre and Department of Neurology, GH Saint-Louis-Lariboisière, Assistance Publique des Hôpitaux de Paris AP HP, Université Paris Denis Diderot and DHU NeuroVasc Sorbonne Paris-Cité, Paris, France

[3]UMR 1161, INSERM, Paris, France

Supplementary Methods

Survival models

The **Cox Proportional Hazards model** (CPH) is commonly used for the study of the relationships between potential predictors and the instantaneous hazard of the event of interest.[1] The Cox model assumes a semi-parametric form for the hazard function:

$$h_i(t|x_i) = h_0(t)\, e^{x_i^T \beta} \tag{1}$$

where $h_i(t)$ is the hazard of event at time t for patient i with his(her) vector of baseline covariates $\boldsymbol{x_i}$, $h_0(t)$ the shared unspecified baseline hazard, and β the vector of parameters to be estimated. Inference is made via the Cox partial likelihood:

$$PL(\beta) = \prod_{i=1}^{N} \left(\frac{e^{x_i^T \beta}}{\sum_{j \in R(t_i)}^{N} e^{x_j^T \beta}} \right)^{\delta_i} \tag{2}$$

where δ_i is the event indicator such that $\delta_i = 1$ if the event occurred and $\delta_i = 0$ if the patient is right-censored, and $R(t)$ is the risk set at time t.

Shrinkage Models. However, it may be relevant to add a regularization term which constrains and shrinks the estimated parameters to increase the generalization capability of the model.[2] Hence, it is common to add an $L1$ penalty, also known as the LASSO (Least Absolute Shrinkage and Selection Operator) penalty, or an $L2$ penalty also named the Ridge penalty, or both of them, called the Elastic Net penalty, which leads to the objective function defined in equation (3):

$$\underset{\beta}{\operatorname{argmax}} \;\; \log PL(\beta) - \alpha \left(r \sum_{j=1}^{p} |\beta_j| + \frac{1-r}{2} \sum_{j=1}^{p} \beta_j^2\right) \tag{3}$$

where α is the regularization intensity, and $r \in [0; 1]$ is the relative weight of the $L1$ and $L2$ regularization. The case when $r = 0$ corresponds to the Ridge Cox Regression and when $r = 1$ to the LASSO Cox Regression. One obvious advantage of LASSO regression over Ridge regression, is that it produces simpler and more interpretable models that incorporate only a reduced set of the predictors. However, neither Ridge regression nor the LASSO will universally dominate the other. The Elastic Net penalty combines both the subset selection property of the LASSO with the regularization strength of the Ridge penalty, which makes it a good improved alternative of the traditional CPH Regression.

Gradient Boosted models (GBM) are ensemble models which combine multiple weak learners sequentially to create an overall strong learner. Multiple base learners slightly better than random guessing are combined in an additive manner to obtain a powerful overall model f of the form:

$$f(\boldsymbol{x}) = \sum_{m=1}^{M} \beta_m g(\boldsymbol{x};\ \theta_m) \tag{4}$$

where M denotes the number of base learners, β_m is a weighting term and g are the base learners with their individual parameters vectors θ_m. For Component-Wise Gradient Boosting, the base learners are component-wise least squares and the objective is to optimize the Cox's partial likelihood loss function.[3] The Gradient Boosting is constructed sequentially, such that it exploits each previous base learner to build the next one. To mitigate overfitting, we employed both a small learning rate to shrink the contribution of each base learner and subsampling, which involves using only a fraction of the training data for fitting individual base learners.

Random Survival Forest (RSF) is a tree-based ensemble model with survival trees used as base learners. Each tree is constructed by successive nodes splitting to maximize the statistical difference in survival patterns, where the quality of a split is measured by the log-rank splitting rule. By contrast to GBM that act sequentially, RSF build several base learners independently. Powerful to detect non-linear relationships, survival tree can easily suffer from overfitting which can lead to poor generalization capability.[4] As a result, combining several survival trees predictions can improve the performance of a single one. RSF ensures individual trees are de-correlated by building each tree on a different bootstrap sample of the original training data (also known as "bagging"), and at each node, only evaluate the split criterion for a randomly selected subset of features and threshold. Furthermore, each node required a minimum number of observations to split further, and each node could be split only if it leaves a minimum number of samples in each of the left and right branches. These constraints avoid the creation of small overly specific branches in the tree. Final predictions are formed by aggregating predictions of individual trees in the ensemble.

Feature selection methods

The univariate Cox Regression is a filter performance-driven method as it consists in fitting a Cox Regression for each feature, record the predictive power of each model according to a chosen metric and keep the top k performing features, with k the arbitrarily fixed desired number of features to keep. Other criteria could have been used in this univariate feature selection setting, such as statistical test and p-values, correlation or mutual information for example. However, as we wanted to maximize the predictive performance of our model, we decided to base this method on the performance metric, as detailed below.

The LASSO method is an intrinsic feature selection method as it performs the selection as a part of the model fitting. Thus, we fitted a LASSO Cox Regression on all features with different regularization intensities such that the greater the intensity, the more coefficients are set to zero. The modified objective function of the Cox Regression with the $L1$ penalty term added is defined as in equation (3) with $r = 1$, and $\alpha \geq 0$ is the regularization intensity. Thus, we selected the hyperparameter α corresponding to the desired number of non-zero coefficients which is chosen from a set of possible values that we defined for each model and which is reported in Supplementary Table S2.

Finally, the Recursive Feature Elimination method is an iterative procedure which consists in recursively fitting a model, ranking the features by an importance attribute and discarding the lowest feature in this ranking. In our case, at each step, we fitted a CPH Regression to the data and the least important feature according to the value of its parameter estimation was discarded. This technique was repeated until the desired number of features to select was reached, as defined in Supplementary Table S2.

Model evaluation metrics

Two time-dependent metrics were used to assess model predictive performance. The time-dependent Brier Score was used to evaluate the overall model performance, calibration as well as discrimination, as it measures how far the predicted probability of remaining event-free up to the time point is to the actual observed event.[5] It is defined in equation (6) as:

$$BS(t) = \frac{1}{n} \sum_{i=1}^{n} I(y_i \leq t \wedge \delta_i = 1) \frac{\left(0 - \hat{\pi}(t|\boldsymbol{x}_i)\right)^2}{\hat{G}(y_i)} + I(y_i > t) \frac{\left(1 - \hat{\pi}(t|\boldsymbol{x}_i)\right)^2}{\hat{G}(t)} \tag{6}$$

where $y_i = \min(t_i, c_i) = \begin{cases} t_i \text{ if } \delta_i = 1 \\ c_i \text{ if } \delta_i = 0 \end{cases}$ is the survival time of the i-th individual with t_i corresponding to the time when the event occurred and c_i the time of censoring, $\hat{\pi}(t|\boldsymbol{x_i})$ is the predicted probability of remaining event-free up to time t for a feature vector $\boldsymbol{x_i}$, and $1/\hat{G}(t)$ is the inverse probability of censoring weight (IPCW), estimated by the Kaplan-Meier estimator. Hence, the Brier Score is bounded in $[0; 1]$, and the lower the score is, the better the model is.

To assess the discrimination power of our model, we used the time-dependent cumulative/dynamic AUC, defined as:

$$\widehat{AUC}(t) = \frac{\sum_{i=1}^{n} \sum_{j=1}^{n} I\left(y_j > t\right) I(y_i \leq t)\, \omega_i\, I\left(\hat{f}\left(\boldsymbol{x}_j\right) \leq \hat{f}(\boldsymbol{x}_i)\right)}{\left(\sum_{i=1}^{n} I(y_i > t)\right)\left(\sum_{i=1}^{n} I(y_i \leq t)\, \omega_i\right)} \tag{7}$$

where $\hat{f}(\boldsymbol{x_i})$ is the risk score of the i-th individual and ω_i the inverse probability of censoring weight. Cumulative cases are all individuals that experienced an event prior to or at time t ($t_i \leq t$) whereas dynamic controls are those with $t_i > t$. The associated cumulative/dynamic AUC quantifies how well a model can distinguish subjects who fail by a given time t from subjects who fail after this time. This metric is also bounded in $[0; 1]$, but here the higher the AUC the better the model is.[6]

As both of these metrics are time-dependent, we arbitrarily decided to set our time of interest at five years from baseline to evaluate the quality of our models.

Supplementary Table S1: Features

This table shows all the features considered in our models. These features are categorized under various headings, including patient identification, medical history, disease history, MRI features, genetic information, clinical examination, biological sampling, and clinical/cognitive/neuropsychological scores.

**Feature category**	**Feature**
Patient identification	Sex
	Inclusion age
	Has high level of education (> 13 years)
Medical history and associated pathology	Pleuropulmonary pathology
	Endocrinal pathology
	Uronephrological pathology
	Digestive pathology
	Osteoarticular pathology
	Head trauma
	Other medical history
	Smoking
	Drinking
	High blood pressure
	Hypercholesterolemia
Disease history	Transient ischemic attack symptom
	Ischemic stroke symptom
	Migraine with aura symptom
	Cognitive disorder symptom
	Balance or walking disorder symptom
	Mental or mood disorder symptom
	Number of strokes before inclusion
	Had at least one stroke before inclusion
	Had a stroke before inclusion with at least one detailed information (type, symptom, date, duration)
	Migraine without aura
	Migraine with aura or isolated aura
	Multiple aura
	Episodic tension headache
	Chronic tension headache
	Other headache
	Episodic or chronic or cluster headache
	Psychiatric disorder
	Number of psychiatric disorders
	Seizure
MRI features	Brain parenchymal fraction
	White matter hyperintensities intraclass correlation coefficient
	Number of lacunes
	Has lacune
	Number of microbleeds
	Has microbleed
Genetic information	EGFr domain 7-34
Clinical examination	Balance or walking disorder symptom
	Visual impairment
	Swallowing disorder
	Hearing impairment
	Urinary problem
	Pyramidal syndrome

	Cerebellar syndrome
	Sensory deficit
	Speech disorder (aphasia)
	Swallowing disorder
	Dysarthria
	Dementia
	Weight
	Systolic blood pressure (mmHg)
	Diastolic blood pressure (mmHg)
Biological sampling	Hemoglobin (g/dL)
	Leucocytes (10e9/L)
	Platelets (10e9/L)
	HDL cholesterol (mmol/L)
	LDL cholesterol (mmol/L)
	Total cholesterol (mmol/L)
	Triglycerides (mmol/L)
	C-reactive protein (mg/L)
	Glycated hemoglobin (%)
	Fasting glucose (mmol/L)
	Fibrinogene (g/L)
	VS (mm)
	Antiphospholipid antibody (UGPL/mL)
Clinical and cognitive/neuropsychological scores	Barthel index
	Rankin index
	Mini-Mental State Examination score
	National Institutes of Health Stroke Scale score
	WAIS-R similitudes
	WAIS-R cubes
	WAIS-R memory span
	WAIS-R reverse memory span
	Mattis Dementia Rating Scale score
	VADAS-cog score
	TMT-A time
	TMT-A errors
	TMT-B-time
	TMT-B errors
	TMTBA global score
	Wisconsin test number of completed categories
	Wisconsin test perseverations
	Wisconsin test failure-to-maintain-set
	Grober and Buschke (GB) immediate recall
	GB free recall 1
	GB cued recall 1
	GB free recall 2
	GB cued recall 2
	GB free recall 3
	GB cued recall 3
	GB free delayed recall
	GB cued delayed recall
	Montgomery-Asberg Depression Rating Scale
	Diagnostic and Statistical Manual of Mental Disorders IV: absence of the 5 axis
	Instrumental Activities of Daily Living: daily care
	Instrumental Activities of Daily Living: basic activity

Supplementary Table S2: Best hyperparameters of the final model

This table shows the best hyperparameters after the 10-folds cross-validation for the final model building.

Methods	Python package	Function	Hyperparameters and values
**Survival models**			
Component-Wise Gradient Boosting	scikit-survival	ComponentwiseGradientBoostingSurvivalAnalysis	number of features after feature selection = 60<br>loss = “coxph”,<br>learning_rate = 0.03,<br>n_estimators = 750,<br>subsample = 0.6
Random Survival Forest	scikit-survival	RandomSurvivalForest	number of features after feature selection = 50<br>n_estimators = 1000,<br>max_depth = 10,<br>min_sample_split = 15,<br>min_samples_leaf = 3,<br>max_features = “log2”,<br>max_samples = 0.6

Supplementary Table S3: Hyperparameter tuning

This table shows the useful functions for model and feature selection. The latter was performed with custom functions, in which the main function is shown in this table.

**Methods**	**Python package**	**Function**	**Hyperparameters and values**
**Survival models**			
CPH	scikit-survival	CoxPHSurvivalAnalysis	number of features after feature selection: [10, 15, 20, 25, 30],
Elastic Net Regression	scikit-survival	CoxnetSurvivalAnalysis	number of features after feature selection: [10, 15, 20, 25, 30],<br>l1_ratio: [0.7, 0.8, 0.9],<br>alphas: [0.005, 0.01, 0.03],<br>fit_baseline_model = True,
Component-Wise Gradient Boosting	scikit-survival	ComponentwiseGradientBoostingSurvivalAnalysis	number of features after feature selection: [50, 60, 70],<br>loss = “coxph”,<br>learning_rate: [0.01, 0.03, 0.05, 0.1]<br>n_estimators: [500, 750, 1000, 1500],<br>subsample: [0.8, 1]
Random Survival Forest	scikit-survival	RandomSurvivalForest	number of features after feature selection: [40,50,60,70],<br>n_estimators: [750,1000,1500,2000],<br>max_depth: [2, 5, 10],<br>min_sample_split: [10, 15],<br>min_samples_leaf: [3,5],<br>max_features = “log2”,<br>max_samples = [0.6, 0.7, 0.8, 0.9]
**Feature selection**			
Univariate Cox scoring	scikit-survival	CoxPHSurvivalAnalysis	
LASSO	scikit-survival	CoxnetSurvivalAnalysis	l1_ratio = 1
RFE	scikit-learn	RFE	estimator = CoxPHSurvivalAnalysis(),<br>step = 1
